# Health Tract, No. 127

**Published:** 1865

**Authors:** 


HEALTH TRACT, No. 127.

### THE ONE SPOT.

One single spot on the fair face of a sheet of the best letter-paper will cause its rejection when the manufacturer assorts it for sale.

In obtaining recruits for the army, a single blemish in the eye, a little defect in the hearing, the loss of a finger or a toe, the slightest limp or halt in the gait, is the one fatal spot which causes rejection, however perfect the health in all other respects.

A faultless specimen of manly vigor offers himself for examination, for the purpose of obtaining an insurance on his life, but at the very first trial of the pulse under the surgeon's finger, the certificate is peremptorily denied, because there is a fatal heart-disease lurking under that fair exterior.

Here is a man who for a lifetime has had uniform good health; never dreamed but that he was perfectly well, but noticed for the first time, an hour before, a little white pimple about the mouth, surrounded with several red ones, giving a dull hurting, causing, however, not the slightest apprehension; but meeting the family physician accidentally on the street, he inquires very carelessly: "What is it?" On a close inspection, the experienced practitioner detects the existence of a "malignant tubercle," which he knows will rapidly spread with a discoloration, and end in death within twenty-four hours! as in the case of Miss M. A. B——, last week; of Mr. Henfield, six months ago; and of Mr. Casy, awhile before that, all of Brooklyn.

These are spots physical and fatal, all! There are moral spots just as fatal to character, health and life itself. I knew a young wife, first at Rockaway, who could boast of family, fortune, education, health, and great personal beauty; fascinating in her conversation, faultless in her intercourse with society, and of a benevolence so hearty and so free, that it was impossible for her neighbors not to love her with their whole hearts. But there was one spot, only one; that not known, even to her husband; she would take opium, and died of its over-use at twenty-three.

I have been delighted by the hour in listening to the recitations and reading the manuscript poetry of Mrs. L—— of Kentucky. Neither beautiful nor ugly, but the spoiled and educated child of a rich father. She had a genius and a power which won all hearts, purely. One morning I learned she was dying, although in perfect health the day before. At intervals of a year, the demon of a drunken debauch! came over her. It killed her husband, one of nature's noblemen. The one spot!

I knew a wife, living yet I think, a model of personal purity, of domestic industry, system, order and thoroughness. A slave to the care for her family of healthful, beautiful children, there was no sacrifice, no self-denial which she was not ever ready to make or practice for their comfort. Her husband, as the world goes, was all that could be desired as to industry, system, temperance, regularity and order. It ought to have been a supremely happy family. It was wretched. The one spot was her insufferable ill-nature. It would be untrue to say she seldom came to the table without some expression of dissatisfaction. In twenty-six successive weeks, during which I daily sat at the same table, she never failed once to emit some venom either against the children, the servants, the food, or the weather, or something else. The whole house was kept in a turmoil, no single day ever passed without it! Her only son was driven to an engine-house, did not sleep at home "once in two years;" thence to the gutter; her daughters married for a home, and she went to an asylum in her old age.

There are many young men with whom you can not help being pleased, frank, courteous, magnanimous and kind; they always meet you with a smile and a welcome, and you know it is cordial and sincere. On inquiry, they "drink." The one spot! It blasts all things else.

That daughter is beautiful, amiable and courteous; in all she says or does, there is nothing to hang an adverse criticism upon. The moment she passes from her father's door, dressed in faultless taste; go to her room, and every article it contains has impressed upon it the one spot of incorrigible sloven.

Let the reader this moment inquire, What spot have I? and begin on the instant to wash it out at any and every sacrifice, for they only who are admitted to the mansions of the blessed are those "not having spot or wrinkle, or any such thing."

